# {*N*′-[1-(5-Bromo-2-oxidophenyl-κ*O*)ethyl­idene]-3-hydroxy-2-naphtho­hydrazidato-κ^2^ *N*′,*O*}dibutyl­tin(IV)

**DOI:** 10.1107/S160053680902457X

**Published:** 2009-07-04

**Authors:** See Mun Lee, Kong Mun Lo, Hapipah Mohd Ali, Seik Weng Ng

**Affiliations:** a Department of Chemistry, University of Malaya, 50603 Kuala Lumpur, Malaysia

## Abstract

The Sn^IV^ atom in the title compound, [Sn(C_4_H_9_)_2_(C_19_H_13_BrN_2_O_3_)], shows a distorted *cis*-C_2_NO_2_Sn trigonal-bipyramidal coordination. Both butyl chains and the naphth­yl­oxy portion are disordered over two sets of sites of equal occupancy.

## Related literature

The dianions of similar *N*′-(2-hydroxy­benzyl­idene)benzohydrazones *O*,*N*,*O*′-chelate to tin in organotin compounds; see: Labib *et al.* (1996 ▶); Samanta *et al.* (2007 ▶).
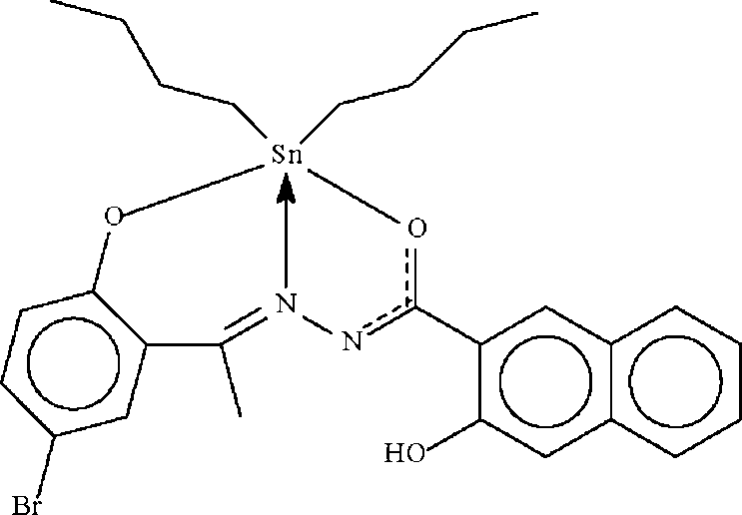


## Experimental

### 

#### Crystal data

[Sn(C_4_H_9_)_2_(C_19_H_13_BrN_2_O_3_)]
*M* *_r_* = 630.14
Monoclinic, 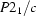

*a* = 14.2649 (2) Å
*b* = 7.2249 (1) Å
*c* = 24.9527 (3) Å
β = 95.483 (1)°
*V* = 2559.92 (6) Å^3^
*Z* = 4
Mo *K*α radiation
μ = 2.59 mm^−1^
*T* = 140 K
0.40 × 0.30 × 0.20 mm

#### Data collection

Bruker SMART APEX diffractometer
Absorption correction: multi-scan (*SADABS*; Sheldrick, 1996 ▶) *T*_min_ = 0.424, *T*_max_ = 0.625
23522 measured reflections
5885 independent reflections
5168 reflections with *I* > 2σ(*I*)
*R*_int_ = 0.031

#### Refinement

*R*[*F* ^2^ > 2σ(*F* ^2^)] = 0.043
*wR*(*F* ^2^) = 0.114
*S* = 1.26
5885 reflections
318 parameters
160 restraints
H-atom parameters constrained
Δρ_max_ = 1.36 e Å^−3^
Δρ_min_ = −1.07 e Å^−3^

### 

Data collection: *APEX2* (Bruker, 2007 ▶); cell refinement: *SAINT* (Bruker, 2007 ▶); data reduction: *SAINT*; program(s) used to solve structure: *SHELXS97* (Sheldrick, 2008 ▶); program(s) used to refine structure: *SHELXL97* (Sheldrick, 2008 ▶); molecular graphics: *X-SEED* (Barbour, 2001 ▶); software used to prepare material for publication: *publCIF* (Westrip, 2009 ▶).

## Supplementary Material

Crystal structure: contains datablocks global, I. DOI: 10.1107/S160053680902457X/xu2545sup1.cif

Structure factors: contains datablocks I. DOI: 10.1107/S160053680902457X/xu2545Isup2.hkl

Additional supplementary materials:  crystallographic information; 3D view; checkCIF report

## Figures and Tables

**Fig. 1.**
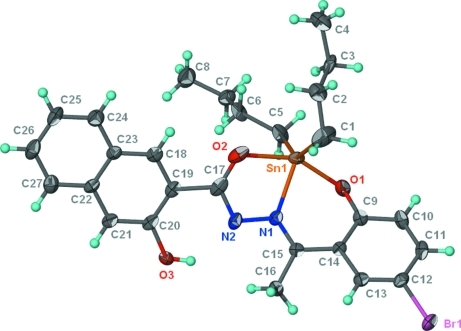
Thermal ellipsoid plot (Barbour, 2001) of Sn(C4H9)2(C19H13BrN2O3) at the 70% probability level. Hydrogen atoms are drawn as spheres of arbitrary radius. One of the disordered components is not shown.

## supplementary crystallographic information

### Experimental

The Schiff base (0.4 g, 1 mmol) from the condensation reaction of 5-bromo-2'-hydroxyacetophenone and 3-hydroxy-2-naphthoic hydrazide and dibutyltin oxide (0.25 g, 1 mmol) were dissolved heated in ethanol (100 ml) until the oxide dissolved completely over an hour. Slow cooling of the filtrate gave the product as yellow crystals.

### Refinement

Both butyl chains are disordered over two positions. One chain is disodered in all four carbon atoms whereas the other is disordered in only the β, γ and δ atoms only. As the occupancy refined to nearly 50:50, this was fixed as exactly 50:50. The C–C distance was tightly restrained to 1.500±0.005 Å and the C···C distance to 2.35±0.01 Å.

The hydroxynaphthyl portion is alo disordered over two positions. The occupancy was also set as 50:50. The naphthyl fused-ring was refined as a rigid ring of 1.39 Å sides. The C17–C19 and C17–C19' distances were restrained to within 0.01 Å of each other, as were the O3–C20 and O3'–C20' distances.

For the chains and fused-ring, the temperature factors of the primed atoms were restrained to those of the unprimed ones; the anisotropic temperature factors were restrained to be nearly isotropic.

Hydrogen atoms were placed at calculated positions (C–H 0.95–0.98 Å) and were treated as riding on their parent atoms, with *U*(H) set to 1.2–1.5*U*_eq_(C). The hydroxy H-atom was similarly treated.

The final difference map had a peak near Sn1 and a hole near Br1.

### Crystal data

**Table:**

[Sn(C_4_H_9_)_2_(C_19_H_13_BrN_2_O_3_)]	*F*(000) = 1264
*M**_r_* = 630.14	*D*_x_ = 1.635 Mg m^−^^3^
Monoclinic, *P*2_1_/*c*	Mo *K*α radiation, λ = 0.71073 Å
Hall symbol: -P 2ybc	Cell parameters from 9057 reflections
*a* = 14.2649 (2) Å	θ = 2.7–30.2°
*b* = 7.2249 (1) Å	µ = 2.59 mm^−^^1^
*c* = 24.9527 (3) Å	*T* = 140 K
β = 95.483 (1)°	Block, yellow
*V* = 2559.92 (6) Å^3^	0.40 × 0.30 × 0.20 mm
*Z* = 4	

### Data collection

**Table:**

Bruker SMART APEX diffractometer	5885 independent reflections
Radiation source: fine-focus sealed tube	5168 reflections with *I* > 2σ(*I*)
graphite	*R*_int_ = 0.031
ω scans	θ_max_ = 27.5°, θ_min_ = 1.6°
Absorption correction: multi-scan (*SADABS*; Sheldrick, 1996)	*h* = −18→18
*T*_min_ = 0.424, *T*_max_ = 0.625	*k* = −9→9
23522 measured reflections	*l* = −32→32

### Refinement

**Table:**

Refinement on *F*^2^	Primary atom site location: structure-invariant direct methods
Least-squares matrix: full	Secondary atom site location: difference Fourier map
*R*[*F*^2^ > 2σ(*F*^2^)] = 0.043	Hydrogen site location: inferred from neighbouring sites
*wR*(*F*^2^) = 0.114	H-atom parameters constrained
*S* = 1.26	*w* = 1/[σ^2^(*F*_o_^2^) + (0.05*P*)^2^ + 5*P*] where *P* = (*F*_o_^2^ + 2*F*_c_^2^)/3
5885 reflections	(Δ/σ)_max_ = 0.001
318 parameters	Δρ_max_ = 1.36 e Å^−^^3^
160 restraints	Δρ_min_ = −1.07 e Å^−^^3^

### Fractional atomic coordinates and isotropic or equivalent isotropic displacement parameters (Å^2^)

**Table:**

	*x*	*y*	*z*	*U*_iso_*/*U*_eq_	Occ. (<1)
Sn1	0.72540 (2)	0.55576 (4)	0.662024 (11)	0.02024 (10)	
Br1	1.03334 (3)	0.81137 (9)	0.906936 (19)	0.03699 (15)	
O1	0.8347 (2)	0.4432 (4)	0.71201 (12)	0.0240 (6)	
O2	0.5866 (2)	0.6711 (4)	0.64649 (12)	0.0292 (7)	
O3	0.4054 (8)	0.5807 (12)	0.7760 (3)	0.0225 (14)	0.50
H3	0.4640	0.5753	0.7838	0.034*	0.50
O3'	0.4054 (8)	0.6330 (12)	0.7628 (3)	0.0225 (14)	0.50
H3'	0.4634	0.6263	0.7723	0.034*	0.50
N1	0.6717 (2)	0.6122 (5)	0.73982 (13)	0.0176 (7)	
N2	0.5737 (2)	0.6253 (5)	0.73690 (15)	0.0221 (7)	
C1	0.8061 (10)	0.775 (2)	0.6303 (3)	0.025 (2)	0.50
H1A	0.7981	0.8877	0.6518	0.029*	0.50
H1B	0.8735	0.7400	0.6354	0.029*	0.50
C2	0.7814 (7)	0.8201 (10)	0.5719 (3)	0.0256 (13)	0.50
H2A	0.7125	0.8383	0.5649	0.031*	0.50
H2B	0.8130	0.9363	0.5628	0.031*	0.50
C3	0.8119 (7)	0.6666 (11)	0.5379 (3)	0.0257 (16)	0.50
H3A	0.7759	0.5531	0.5447	0.031*	0.50
H3B	0.8796	0.6406	0.5475	0.031*	0.50
C4	0.7958 (8)	0.7182 (15)	0.4797 (3)	0.0352 (17)	0.50
H4A	0.7913	0.6056	0.4577	0.053*	0.50
H4B	0.8484	0.7941	0.4698	0.053*	0.50
H4C	0.7370	0.7886	0.4734	0.053*	0.50
C1'	0.7823 (9)	0.813 (2)	0.6364 (3)	0.025 (2)	0.50
H1'A	0.7295	0.8995	0.6270	0.029*	0.50
H1'B	0.8221	0.8671	0.6671	0.029*	0.50
C2'	0.8397 (6)	0.7984 (12)	0.5893 (3)	0.0256 (13)	0.50
H2'A	0.8645	0.9220	0.5810	0.031*	0.50
H2'B	0.8939	0.7150	0.5985	0.031*	0.50
C3'	0.7811 (5)	0.7253 (14)	0.5414 (3)	0.0257 (16)	0.50
H3'A	0.7284	0.8110	0.5310	0.031*	0.50
H3'B	0.7546	0.6033	0.5498	0.031*	0.50
C4'	0.8425 (7)	0.7066 (15)	0.4961 (3)	0.0352 (17)	0.50
H4'A	0.8038	0.6705	0.4632	0.053*	0.50
H4'B	0.8905	0.6118	0.5052	0.053*	0.50
H4'C	0.8732	0.8253	0.4903	0.053*	0.50
C5	0.6858 (3)	0.2933 (7)	0.62678 (19)	0.0312 (11)	
H5A	0.7358	0.2511	0.6047	0.037*	0.50
H5B	0.6811	0.2016	0.6559	0.037*	0.50
H5C	0.7234	0.2764	0.5958	0.037*	0.50
H5D	0.7085	0.1997	0.6539	0.037*	0.50
C6	0.5922 (5)	0.2992 (10)	0.5915 (3)	0.0248 (16)	0.50
H6A	0.5426	0.3498	0.6125	0.030*	0.50
H6B	0.5980	0.3822	0.5604	0.030*	0.50
C7	0.5645 (5)	0.1108 (10)	0.5717 (3)	0.0260 (13)	0.50
H7A	0.5608	0.0266	0.6027	0.031*	0.50
H7B	0.6128	0.0620	0.5494	0.031*	0.50
C8	0.4706 (6)	0.1169 (14)	0.5387 (4)	0.0367 (16)	0.50
H8A	0.4533	−0.0080	0.5260	0.055*	0.50
H8B	0.4747	0.1987	0.5077	0.055*	0.50
H8C	0.4228	0.1639	0.5609	0.055*	0.50
C6'	0.5857 (4)	0.2476 (13)	0.6092 (3)	0.0248 (16)	0.50
H6'A	0.5764	0.1119	0.6074	0.030*	0.50
H6'B	0.5434	0.3002	0.6344	0.030*	0.50
C7'	0.5664 (5)	0.3320 (12)	0.5547 (3)	0.0260 (13)	0.50
H7'A	0.6073	0.2758	0.5293	0.031*	0.50
H7'B	0.5788	0.4668	0.5565	0.031*	0.50
C8'	0.4646 (5)	0.2961 (15)	0.5365 (4)	0.0367 (16)	0.50
H8'A	0.4474	0.3623	0.5027	0.055*	0.50
H8'B	0.4253	0.3395	0.5640	0.055*	0.50
H8'C	0.4549	0.1630	0.5307	0.055*	0.50
C9	0.8749 (3)	0.5319 (5)	0.75490 (17)	0.0188 (8)	
C10	0.9737 (3)	0.5253 (6)	0.76486 (19)	0.0226 (9)	
H10	1.0092	0.4619	0.7402	0.027*	
C11	1.0200 (3)	0.6084 (6)	0.80931 (18)	0.0214 (8)	
H11	1.0868	0.6038	0.8152	0.026*	
C12	0.9682 (3)	0.6989 (6)	0.84552 (16)	0.0212 (8)	
C13	0.8721 (3)	0.7089 (6)	0.83758 (16)	0.0195 (8)	
H13	0.8384	0.7733	0.8629	0.023*	
C14	0.8220 (3)	0.6247 (5)	0.79235 (15)	0.0163 (7)	
C15	0.7191 (3)	0.6337 (5)	0.78681 (15)	0.0166 (7)	
C16	0.6671 (3)	0.6700 (6)	0.83560 (16)	0.0205 (8)	
H16A	0.6065	0.6052	0.8317	0.031*	
H16B	0.6563	0.8033	0.8389	0.031*	
H16C	0.7048	0.6252	0.8679	0.031*	
C17	0.5374 (3)	0.6540 (6)	0.68713 (18)	0.0249 (9)	
C18	0.3848 (2)	0.7007 (9)	0.63169 (18)	0.0194 (15)	0.50
H18	0.4233	0.7234	0.6034	0.023*	0.50
C19	0.4258 (2)	0.6540 (9)	0.6827 (2)	0.0189 (14)	0.50
C20	0.3694 (3)	0.6209 (9)	0.72411 (17)	0.0185 (15)	0.50
C21	0.2721 (3)	0.6345 (7)	0.71459 (14)	0.0192 (14)	0.50
H21	0.2335	0.6119	0.7429	0.023*	0.50
C22	0.2310 (2)	0.6813 (5)	0.66361 (14)	0.0182 (14)	0.50
C23	0.2874 (2)	0.7144 (6)	0.62216 (13)	0.0174 (15)	0.50
C24	0.2464 (3)	0.7612 (8)	0.57118 (14)	0.0258 (15)	0.50
H24	0.2849	0.7838	0.5429	0.031*	0.50
C25	0.1490 (3)	0.7748 (8)	0.56165 (17)	0.0278 (15)	0.50
H25	0.1210	0.8068	0.5268	0.033*	0.50
C26	0.0927 (2)	0.7417 (8)	0.6031 (2)	0.0266 (15)	0.50
H26	0.0261	0.7511	0.5966	0.032*	0.50
C27	0.1337 (2)	0.6950 (7)	0.65408 (19)	0.0240 (13)	0.50
H27	0.0952	0.6724	0.6824	0.029*	0.50
C18'	0.4052 (2)	0.7136 (9)	0.61716 (18)	0.0194 (15)	0.50
H18'	0.4487	0.7251	0.5908	0.023*	0.50
C19'	0.4376 (2)	0.6775 (10)	0.6704 (2)	0.0189 (14)	0.50
C20'	0.3740 (3)	0.6606 (8)	0.70904 (16)	0.0185 (15)	0.50
C21'	0.2780 (3)	0.6799 (7)	0.69436 (14)	0.0192 (14)	0.50
H21'	0.2346	0.6684	0.7207	0.023*	0.50
C22'	0.2456 (2)	0.7160 (5)	0.64107 (14)	0.0182 (14)	0.50
C23'	0.3092 (2)	0.7328 (6)	0.60247 (14)	0.0174 (15)	0.50
C24'	0.2768 (3)	0.7690 (8)	0.54918 (14)	0.0258 (15)	0.50
H24'	0.3203	0.7805	0.5228	0.031*	0.50
C25'	0.1809 (3)	0.7882 (8)	0.53450 (16)	0.0278 (15)	0.50
H25'	0.1587	0.8129	0.4981	0.033*	0.50
C26'	0.1173 (3)	0.7714 (8)	0.5731 (2)	0.0266 (15)	0.50
H26B	0.0517	0.7846	0.5631	0.032*	0.50
C27'	0.1497 (2)	0.7353 (7)	0.62638 (19)	0.0240 (13)	0.50
H27'	0.1062	0.7237	0.6528	0.029*	0.50

### Atomic displacement parameters (Å^2^)

**Table:**

	*U*^11^	*U*^22^	*U*^33^	*U*^12^	*U*^13^	*U*^23^
Sn1	0.02501 (16)	0.02065 (15)	0.01516 (15)	−0.00916 (11)	0.00247 (10)	−0.00324 (10)
Br1	0.0226 (2)	0.0629 (4)	0.0237 (2)	−0.0061 (2)	−0.00660 (17)	−0.0052 (2)
O1	0.0206 (15)	0.0243 (15)	0.0275 (16)	−0.0007 (12)	0.0045 (12)	−0.0100 (12)
O2	0.0418 (19)	0.0202 (15)	0.0231 (16)	0.0017 (14)	−0.0097 (14)	0.0005 (12)
O3	0.0197 (16)	0.028 (4)	0.020 (3)	0.001 (3)	0.003 (2)	0.004 (2)
O3'	0.0197 (16)	0.028 (4)	0.020 (3)	0.001 (3)	0.003 (2)	0.004 (2)
N1	0.0179 (16)	0.0150 (15)	0.0195 (16)	0.0031 (13)	−0.0009 (13)	−0.0006 (13)
N2	0.0181 (17)	0.0212 (17)	0.0256 (18)	0.0063 (14)	−0.0049 (14)	−0.0086 (14)
C1	0.021 (7)	0.021 (7)	0.033 (3)	−0.007 (4)	0.005 (3)	−0.002 (3)
C2	0.029 (3)	0.022 (2)	0.027 (3)	−0.002 (2)	0.006 (2)	0.003 (2)
C3	0.022 (3)	0.027 (3)	0.028 (2)	0.002 (2)	0.002 (2)	0.002 (2)
C4	0.038 (3)	0.035 (3)	0.033 (3)	0.003 (3)	0.007 (3)	0.003 (2)
C1'	0.021 (7)	0.021 (7)	0.033 (3)	−0.007 (4)	0.005 (3)	−0.002 (3)
C2'	0.029 (3)	0.022 (2)	0.027 (3)	−0.002 (2)	0.006 (2)	0.003 (2)
C3'	0.022 (3)	0.027 (3)	0.028 (2)	0.002 (2)	0.002 (2)	0.002 (2)
C4'	0.038 (3)	0.035 (3)	0.033 (3)	0.003 (3)	0.007 (3)	0.003 (2)
C5	0.026 (2)	0.032 (2)	0.038 (3)	−0.0160 (19)	0.0135 (19)	−0.020 (2)
C6	0.023 (2)	0.022 (3)	0.031 (3)	−0.013 (2)	0.012 (2)	−0.004 (2)
C7	0.027 (3)	0.026 (2)	0.024 (2)	−0.002 (2)	0.001 (2)	−0.003 (2)
C8	0.036 (3)	0.038 (3)	0.036 (3)	−0.001 (2)	0.002 (2)	−0.004 (2)
C6'	0.023 (2)	0.022 (3)	0.031 (3)	−0.013 (2)	0.012 (2)	−0.004 (2)
C7'	0.027 (3)	0.026 (2)	0.024 (2)	−0.002 (2)	0.001 (2)	−0.003 (2)
C8'	0.036 (3)	0.038 (3)	0.036 (3)	−0.001 (2)	0.002 (2)	−0.004 (2)
C9	0.021 (2)	0.0127 (18)	0.023 (2)	0.0001 (15)	0.0037 (15)	−0.0008 (15)
C10	0.018 (2)	0.0156 (19)	0.035 (2)	0.0025 (15)	0.0067 (17)	−0.0023 (16)
C11	0.0153 (19)	0.0156 (18)	0.033 (2)	0.0008 (15)	−0.0012 (16)	0.0076 (16)
C12	0.023 (2)	0.0216 (19)	0.0184 (19)	−0.0025 (16)	−0.0033 (15)	0.0051 (16)
C13	0.023 (2)	0.0181 (19)	0.0175 (19)	0.0013 (15)	0.0009 (15)	0.0026 (15)
C14	0.0187 (19)	0.0130 (17)	0.0169 (18)	0.0000 (14)	0.0003 (14)	0.0015 (14)
C15	0.021 (2)	0.0110 (17)	0.0177 (18)	0.0021 (14)	0.0021 (15)	0.0013 (14)
C16	0.0183 (19)	0.022 (2)	0.022 (2)	−0.0003 (16)	0.0039 (15)	−0.0018 (16)
C17	0.031 (2)	0.0129 (19)	0.029 (2)	0.0060 (16)	−0.0079 (18)	−0.0073 (16)
C18	0.022 (3)	0.017 (2)	0.019 (3)	0.004 (2)	0.002 (3)	−0.002 (2)
C19	0.022 (2)	0.012 (2)	0.022 (3)	0.0022 (19)	−0.002 (2)	−0.001 (2)
C20	0.023 (2)	0.012 (3)	0.019 (3)	0.007 (2)	−0.004 (2)	0.000 (3)
C21	0.022 (2)	0.017 (3)	0.018 (3)	0.005 (2)	0.000 (3)	−0.001 (2)
C22	0.022 (3)	0.015 (3)	0.018 (3)	0.000 (2)	−0.002 (3)	−0.001 (2)
C23	0.019 (3)	0.016 (2)	0.016 (3)	0.002 (2)	0.000 (2)	−0.003 (2)
C24	0.026 (3)	0.026 (3)	0.025 (3)	−0.001 (3)	0.001 (2)	−0.001 (3)
C25	0.024 (3)	0.029 (3)	0.028 (3)	−0.004 (3)	−0.007 (2)	0.001 (3)
C26	0.022 (3)	0.024 (3)	0.032 (3)	0.003 (2)	−0.006 (3)	−0.009 (3)
C27	0.022 (3)	0.021 (3)	0.028 (3)	−0.003 (2)	0.000 (3)	−0.003 (2)
C18'	0.022 (3)	0.017 (2)	0.019 (3)	0.004 (2)	0.002 (3)	−0.002 (2)
C19'	0.022 (2)	0.012 (2)	0.022 (3)	0.0022 (19)	−0.002 (2)	−0.001 (2)
C20'	0.023 (2)	0.012 (3)	0.019 (3)	0.007 (2)	−0.004 (2)	0.000 (3)
C21'	0.022 (2)	0.017 (3)	0.018 (3)	0.005 (2)	0.000 (3)	−0.001 (2)
C22'	0.022 (3)	0.015 (3)	0.018 (3)	0.000 (2)	−0.002 (3)	−0.001 (2)
C23'	0.019 (3)	0.016 (2)	0.016 (3)	0.002 (2)	0.000 (2)	−0.003 (2)
C24'	0.026 (3)	0.026 (3)	0.025 (3)	−0.001 (3)	0.001 (2)	−0.001 (3)
C25'	0.024 (3)	0.029 (3)	0.028 (3)	−0.004 (3)	−0.007 (2)	0.001 (3)
C26'	0.022 (3)	0.024 (3)	0.032 (3)	0.003 (2)	−0.006 (3)	−0.009 (3)
C27'	0.022 (3)	0.021 (3)	0.028 (3)	−0.003 (2)	0.000 (3)	−0.003 (2)

### Geometric parameters (Å, °)

**Table:**

Sn1—O1	2.068 (3)	C6'—H6'B	0.9900
Sn1—C5	2.143 (4)	C7'—C8'	1.502 (5)
Sn1—C1'	2.149 (18)	C7'—H7'A	0.9900
Sn1—O2	2.150 (3)	C7'—H7'B	0.9900
Sn1—C1	2.150 (18)	C8'—H8'A	0.9800
Sn1—N1	2.192 (3)	C8'—H8'B	0.9800
Br1—C12	1.897 (4)	C8'—H8'C	0.9800
O1—C9	1.329 (5)	C9—C10	1.409 (6)
O2—C17	1.292 (6)	C9—C14	1.423 (5)
O3—C20	1.377 (7)	C10—C11	1.374 (6)
O3—H3	0.8400	C10—H10	0.9500
O3'—C20'	1.388 (7)	C11—C12	1.385 (6)
O3'—H3'	0.8400	C11—H11	0.9500
N1—C15	1.306 (5)	C12—C13	1.369 (6)
N1—N2	1.396 (5)	C13—C14	1.414 (6)
N2—C17	1.315 (6)	C13—H13	0.9500
C1—C2	1.503 (5)	C14—C15	1.462 (5)
C1—H1A	0.9900	C15—C16	1.508 (5)
C1—H1B	0.9900	C16—H16A	0.9800
C2—C3	1.486 (5)	C16—H16B	0.9800
C2—H2A	0.9900	C16—H16C	0.9800
C2—H2B	0.9900	C17—C19'	1.455 (5)
C3—C4	1.497 (5)	C17—C19	1.586 (6)
C3—H3A	0.9900	C18—C19	1.3900
C3—H3B	0.9900	C18—C23	1.3900
C4—H4A	0.9800	C18—H18	0.9500
C4—H4B	0.9800	C19—C20	1.3900
C4—H4C	0.9800	C20—C21	1.3900
C1'—C2'	1.498 (5)	C21—C22	1.3900
C1'—H1'A	0.9900	C21—H21	0.9500
C1'—H1'B	0.9900	C22—C23	1.3900
C2'—C3'	1.489 (5)	C22—C27	1.3900
C2'—H2'A	0.9900	C23—C24	1.3900
C2'—H2'B	0.9900	C24—C25	1.3900
C3'—C4'	1.501 (5)	C24—H24	0.9500
C3'—H3'A	0.9900	C25—C26	1.3900
C3'—H3'B	0.9900	C25—H25	0.9500
C4'—H4'A	0.9800	C26—C27	1.3900
C4'—H4'B	0.9800	C26—H26	0.9500
C4'—H4'C	0.9800	C27—H27	0.9500
C5—C6'	1.491 (5)	C18'—C19'	1.3900
C5—C6	1.529 (5)	C18'—C23'	1.3900
C5—H5A	0.9900	C18'—H18'	0.9500
C5—H5B	0.9900	C19'—C20'	1.3900
C5—H5C	0.9901	C20'—C21'	1.3900
C5—H5D	0.9901	C21'—C22'	1.3900
C6—C7	1.488 (5)	C21'—H21'	0.9500
C6—H6A	0.9900	C22'—C23'	1.3900
C6—H6B	0.9900	C22'—C27'	1.3900
C7—C8	1.503 (5)	C23'—C24'	1.3900
C7—H7A	0.9900	C24'—C25'	1.3900
C7—H7B	0.9900	C24'—H24'	0.9500
C8—H8A	0.9800	C25'—C26'	1.3900
C8—H8B	0.9800	C25'—H25'	0.9500
C8—H8C	0.9800	C26'—C27'	1.3900
C6'—C7'	1.490 (5)	C26'—H26B	0.9500
C6'—H6'A	0.9900	C27'—H27'	0.9500
			
O1—Sn1—C5	93.07 (15)	C6'—C7'—H7'A	110.3
O1—Sn1—C1'	103.6 (3)	C8'—C7'—H7'A	110.3
C5—Sn1—C1'	137.3 (3)	C6'—C7'—H7'B	110.3
O1—Sn1—O2	152.85 (12)	C8'—C7'—H7'B	110.3
C5—Sn1—O2	93.84 (14)	H7'A—C7'—H7'B	108.5
C1'—Sn1—O2	88.8 (3)	O1—C9—C10	118.2 (4)
O1—Sn1—C1	96.4 (3)	O1—C9—C14	122.8 (4)
C5—Sn1—C1	129.0 (3)	C10—C9—C14	119.0 (4)
C1'—Sn1—C1	12.7 (4)	C11—C10—C9	121.6 (4)
O2—Sn1—C1	99.5 (3)	C11—C10—H10	119.2
O1—Sn1—N1	81.16 (12)	C9—C10—H10	119.2
C5—Sn1—N1	115.29 (16)	C10—C11—C12	119.2 (4)
C1'—Sn1—N1	106.0 (2)	C10—C11—H11	120.4
O2—Sn1—N1	72.15 (12)	C12—C11—H11	120.4
C1—Sn1—N1	115.7 (3)	C13—C12—C11	121.4 (4)
C9—O1—Sn1	122.5 (3)	C13—C12—Br1	120.1 (3)
C17—O2—Sn1	112.4 (3)	C11—C12—Br1	118.6 (3)
C20—O3—H3	120.0	C12—C13—C14	121.0 (4)
C20'—O3'—H3'	120.0	C12—C13—H13	119.5
C15—N1—N2	118.0 (3)	C14—C13—H13	119.5
C15—N1—Sn1	128.5 (3)	C13—C14—C9	117.9 (4)
N2—N1—Sn1	113.5 (2)	C13—C14—C15	118.8 (4)
C17—N2—N1	111.2 (4)	C9—C14—C15	123.3 (4)
C2—C1—Sn1	115.7 (10)	N1—C15—C14	120.6 (3)
C2—C1—H1A	108.4	N1—C15—C16	119.5 (4)
Sn1—C1—H1A	108.4	C14—C15—C16	119.9 (3)
C2—C1—H1B	108.4	C15—C16—H16A	109.5
Sn1—C1—H1B	108.4	C15—C16—H16B	109.5
H1A—C1—H1B	107.4	H16A—C16—H16B	109.5
C3—C2—C1	109.6 (6)	C15—C16—H16C	109.5
C3—C2—H2A	109.8	H16A—C16—H16C	109.5
C1—C2—H2A	109.8	H16B—C16—H16C	109.5
C3—C2—H2B	109.8	O2—C17—N2	124.2 (4)
C1—C2—H2B	109.8	O2—C17—C19'	110.7 (4)
H2A—C2—H2B	108.2	N2—C17—C19'	125.1 (4)
C2—C3—C4	110.0 (6)	O2—C17—C19	124.1 (4)
C2—C3—H3A	109.7	N2—C17—C19	111.6 (4)
C4—C3—H3A	109.7	C19—C18—C23	120.0
C2—C3—H3B	109.7	C19—C18—H18	120.0
C4—C3—H3B	109.7	C23—C18—H18	120.0
H3A—C3—H3B	108.2	C20—C19—C18	120.0
C2'—C1'—Sn1	115.0 (9)	C20—C19—C17	126.6 (3)
C2'—C1'—H1'A	108.5	C18—C19—C17	113.3 (3)
Sn1—C1'—H1'A	108.5	O3—C20—C19	123.1 (5)
C2'—C1'—H1'B	108.5	O3—C20—C21	116.9 (5)
Sn1—C1'—H1'B	108.5	C19—C20—C21	120.0
H1'A—C1'—H1'B	107.5	C20—C21—C22	120.0
C3'—C2'—C1'	110.4 (6)	C20—C21—H21	120.0
C3'—C2'—H2'A	109.6	C22—C21—H21	120.0
C1'—C2'—H2'A	109.6	C23—C22—C21	120.0
C3'—C2'—H2'B	109.6	C23—C22—C27	120.0
C1'—C2'—H2'B	109.6	C21—C22—C27	120.0
H2'A—C2'—H2'B	108.1	C24—C23—C22	120.0
C2'—C3'—C4'	108.3 (6)	C24—C23—C18	120.0
C2'—C3'—H3'A	110.0	C22—C23—C18	120.0
C4'—C3'—H3'A	110.0	C23—C24—C25	120.0
C2'—C3'—H3'B	110.0	C23—C24—H24	120.0
C4'—C3'—H3'B	110.0	C25—C24—H24	120.0
H3'A—C3'—H3'B	108.4	C24—C25—C26	120.0
C3'—C4'—H4'A	109.5	C24—C25—H25	120.0
C3'—C4'—H4'B	109.5	C26—C25—H25	120.0
H4'A—C4'—H4'B	109.5	C27—C26—C25	120.0
C3'—C4'—H4'C	109.5	C27—C26—H26	120.0
H4'A—C4'—H4'C	109.5	C25—C26—H26	120.0
H4'B—C4'—H4'C	109.5	C26—C27—C22	120.0
C6'—C5—Sn1	121.6 (5)	C26—C27—H27	120.0
C6—C5—Sn1	113.2 (4)	C22—C27—H27	120.0
C6'—C5—H5A	119.3	C19'—C18'—C23'	120.0
C6—C5—H5A	108.9	C19'—C18'—H18'	120.0
Sn1—C5—H5A	108.9	C23'—C18'—H18'	120.0
C6—C5—H5B	108.9	C20'—C19'—C18'	120.0
Sn1—C5—H5B	108.9	C20'—C19'—C17	118.5 (3)
H5A—C5—H5B	107.8	C18'—C19'—C17	121.5 (3)
C6'—C5—H5C	108.5	O3'—C20'—C19'	120.7 (6)
Sn1—C5—H5C	106.8	O3'—C20'—C21'	119.2 (6)
C6'—C5—H5D	107.0	C19'—C20'—C21'	120.0
Sn1—C5—H5D	105.5	C20'—C21'—C22'	120.0
H5C—C5—H5D	106.6	C20'—C21'—H21'	120.0
C7—C6—C5	110.8 (5)	C22'—C21'—H21'	120.0
C7—C6—H6A	109.5	C23'—C22'—C21'	120.0
C5—C6—H6A	109.5	C23'—C22'—C27'	120.0
C7—C6—H6B	109.5	C21'—C22'—C27'	120.0
C5—C6—H6B	109.5	C24'—C23'—C22'	120.0
H6A—C6—H6B	108.1	C24'—C23'—C18'	120.0
C6—C7—C8	110.5 (6)	C22'—C23'—C18'	120.0
C6—C7—H7A	109.6	C23'—C24'—C25'	120.0
C8—C7—H7A	109.6	C23'—C24'—H24'	120.0
C6—C7—H7B	109.6	C25'—C24'—H24'	120.0
C8—C7—H7B	109.6	C24'—C25'—C26'	120.0
H7A—C7—H7B	108.1	C24'—C25'—H25'	120.0
C7'—C6'—C5	105.5 (5)	C26'—C25'—H25'	120.0
C7'—C6'—H6'A	110.6	C27'—C26'—C25'	120.0
C5—C6'—H6'A	110.6	C27'—C26'—H26B	120.0
C7'—C6'—H6'B	110.6	C25'—C26'—H26B	120.0
C5—C6'—H6'B	110.6	C26'—C27'—C22'	120.0
H6'A—C6'—H6'B	108.8	C26'—C27'—H27'	120.0
C6'—C7'—C8'	107.2 (5)	C22'—C27'—H27'	120.0
			
C5—Sn1—O1—C9	−162.9 (3)	Sn1—N1—C15—C16	−179.4 (3)
C1'—Sn1—O1—C9	56.7 (4)	C13—C14—C15—N1	158.4 (4)
O2—Sn1—O1—C9	−58.3 (4)	C9—C14—C15—N1	−22.9 (6)
C1—Sn1—O1—C9	67.3 (4)	C13—C14—C15—C16	−20.9 (5)
N1—Sn1—O1—C9	−47.8 (3)	C9—C14—C15—C16	157.8 (4)
O1—Sn1—O2—C17	−10.0 (4)	Sn1—O2—C17—N2	20.2 (5)
C5—Sn1—O2—C17	94.4 (3)	Sn1—O2—C17—C19'	−161.9 (4)
C1'—Sn1—O2—C17	−128.3 (4)	Sn1—O2—C17—C19	−156.3 (4)
C1—Sn1—O2—C17	−135.1 (4)	N1—N2—C17—O2	−1.2 (6)
N1—Sn1—O2—C17	−21.0 (3)	N1—N2—C17—C19'	−178.8 (5)
O1—Sn1—N1—C15	26.0 (3)	N1—N2—C17—C19	175.7 (4)
C5—Sn1—N1—C15	115.2 (3)	C23—C18—C19—C20	0.0
C1'—Sn1—N1—C15	−75.7 (5)	C23—C18—C19—C17	−177.2 (4)
O2—Sn1—N1—C15	−159.1 (4)	O2—C17—C19—C20	174.3 (3)
C1—Sn1—N1—C15	−66.9 (5)	N2—C17—C19—C20	−2.6 (6)
O1—Sn1—N1—N2	−153.8 (3)	C19'—C17—C19—C20	−164 (2)
C5—Sn1—N1—N2	−64.6 (3)	O2—C17—C19—C18	−8.7 (5)
C1'—Sn1—N1—N2	104.5 (4)	N2—C17—C19—C18	174.4 (3)
O2—Sn1—N1—N2	21.1 (3)	C19'—C17—C19—C18	12.8 (18)
C1—Sn1—N1—N2	113.3 (4)	C18—C19—C20—O3	−177.5 (6)
C15—N1—N2—C17	161.9 (4)	C17—C19—C20—O3	−0.7 (6)
Sn1—N1—N2—C17	−18.2 (4)	C18—C19—C20—C21	0.0
O1—Sn1—C1—C2	143.1 (6)	C17—C19—C20—C21	176.8 (5)
C5—Sn1—C1—C2	43.9 (8)	O3—C20—C21—C22	177.7 (6)
C1'—Sn1—C1—C2	−92 (3)	C19—C20—C21—C22	0.0
O2—Sn1—C1—C2	−59.0 (7)	C20—C21—C22—C23	0.0
N1—Sn1—C1—C2	−133.7 (6)	C20—C21—C22—C27	180.0
Sn1—C1—C2—C3	−70.2 (9)	C21—C22—C23—C24	180.0
C1—C2—C3—C4	−174.7 (10)	C27—C22—C23—C24	0.0
O1—Sn1—C1'—C2'	84.1 (7)	C21—C22—C23—C18	0.0
C5—Sn1—C1'—C2'	−26.0 (10)	C27—C22—C23—C18	180.0
O2—Sn1—C1'—C2'	−120.3 (7)	C19—C18—C23—C24	180.0
C1—Sn1—C1'—C2'	27.5 (19)	C19—C18—C23—C22	0.0
N1—Sn1—C1'—C2'	168.6 (6)	C22—C23—C24—C25	0.0
Sn1—C1'—C2'—C3'	60.8 (10)	C18—C23—C24—C25	180.0
C1'—C2'—C3'—C4'	−177.9 (10)	C23—C24—C25—C26	0.0
O1—Sn1—C5—C6'	146.5 (4)	C24—C25—C26—C27	0.0
C1'—Sn1—C5—C6'	−99.6 (6)	C25—C26—C27—C22	0.0
O2—Sn1—C5—C6'	−7.3 (4)	C23—C22—C27—C26	0.0
C1—Sn1—C5—C6'	−112.8 (5)	C21—C22—C27—C26	180.0
N1—Sn1—C5—C6'	64.8 (5)	C23'—C18'—C19'—C20'	0.0
O1—Sn1—C5—C6	170.1 (5)	C23'—C18'—C19'—C17	179.2 (5)
C1'—Sn1—C5—C6	−76.0 (7)	O2—C17—C19'—C20'	179.0 (3)
O2—Sn1—C5—C6	16.4 (5)	N2—C17—C19'—C20'	−3.0 (7)
C1—Sn1—C5—C6	−89.1 (6)	C19—C17—C19'—C20'	18.0 (17)
N1—Sn1—C5—C6	88.4 (5)	O2—C17—C19'—C18'	−0.1 (5)
C6'—C5—C6—C7	−58.2 (13)	N2—C17—C19'—C18'	177.8 (3)
Sn1—C5—C6—C7	−175.5 (6)	C19—C17—C19'—C18'	−161 (2)
C5—C6—C7—C8	177.9 (7)	C18'—C19'—C20'—O3'	−177.0 (6)
C6—C5—C6'—C7'	8.5 (11)	C17—C19'—C20'—O3'	3.8 (6)
Sn1—C5—C6'—C7'	82.3 (7)	C18'—C19'—C20'—C21'	0.0
C5—C6'—C7'—C8'	−177.6 (7)	C17—C19'—C20'—C21'	−179.2 (5)
Sn1—O1—C9—C10	−137.7 (3)	O3'—C20'—C21'—C22'	177.1 (6)
Sn1—O1—C9—C14	45.5 (5)	C19'—C20'—C21'—C22'	0.0
O1—C9—C10—C11	−177.8 (4)	C20'—C21'—C22'—C23'	0.0
C14—C9—C10—C11	−1.0 (6)	C20'—C21'—C22'—C27'	180.0
C9—C10—C11—C12	0.8 (6)	C21'—C22'—C23'—C24'	180.0
C10—C11—C12—C13	−0.7 (6)	C27'—C22'—C23'—C24'	0.0
C10—C11—C12—Br1	179.6 (3)	C21'—C22'—C23'—C18'	0.0
C11—C12—C13—C14	0.8 (6)	C27'—C22'—C23'—C18'	180.0
Br1—C12—C13—C14	−179.4 (3)	C19'—C18'—C23'—C24'	180.0
C12—C13—C14—C9	−1.0 (6)	C19'—C18'—C23'—C22'	0.0
C12—C13—C14—C15	177.8 (4)	C22'—C23'—C24'—C25'	0.0
O1—C9—C14—C13	177.8 (4)	C18'—C23'—C24'—C25'	180.0
C10—C9—C14—C13	1.1 (6)	C23'—C24'—C25'—C26'	0.0
O1—C9—C14—C15	−1.0 (6)	C24'—C25'—C26'—C27'	0.0
C10—C9—C14—C15	−177.7 (4)	C25'—C26'—C27'—C22'	0.0
N2—N1—C15—C14	−178.9 (3)	C23'—C22'—C27'—C26'	0.0
Sn1—N1—C15—C14	1.2 (5)	C21'—C22'—C27'—C26'	180.0
N2—N1—C15—C16	0.4 (5)		

### Hydrogen-bond geometry (Å, °)

**Table:**

*D*—H···*A*	*D*—H	H···*A*	*D*···*A*	*D*—H···*A*
O3—H3···N2	0.84	2.07	2.696 (11)	131
O3'—H3'···N2	0.84	1.88	2.546 (12)	136

## References

[bb1] Barbour, L. J. (2001). *J. Supramol. Chem.* **1**, 189–191.

[bb2] Bruker (2007). *APEX2* and *SAINT* Bruker AXS Inc., Madison, Wisconsin, USA.

[bb3] Labib, L., Khalil, T. E., Iskander, M. F. & Refaat, L. S. (1996). *Polyhedron*, **21**, 3697–3707.

[bb4] Samanta, B., Chakraborty, J., Dey, D. K. & Mitra, S. (2007). *Struct. Chem.* **18**, 287–297.

[bb5] Sheldrick, G. M. (1996). *SADABS* University of Göttingen, Germany.

[bb6] Sheldrick, G. M. (2008). *Acta Cryst.* A**64**, 112–122. 10.1107/S0108767307043930 18156677

[bb7] Westrip, S. P. (2009). *publCIF* In preparation.

